# Imaging Findings of Congenital Distal Interphalangeal Joint Dysplasia in a 3‐Month‐Old Friesian Foal

**DOI:** 10.1111/vru.70076

**Published:** 2025-08-19

**Authors:** Emmie J. M. Giessen, Emanuel K. L. Stas, Guy C. M. Grinwis, Stefanie Veraa

**Affiliations:** ^1^ Diagnostic Imaging Department of Clinical Sciences Faculty of Veterinary Medicine Utrecht University Utrecht The Netherlands; ^2^ Equine Clinic Department of Surgery and Radiology Freie Universität Berlin Berlin Germany; ^3^ Department of Biomolecular Health Sciences Faculty of Veterinary Medicine Utrecht University Utrecht The Netherlands

**Keywords:** coffin joint, developmental, equine, osteochondrosis

## Abstract

A 3‐month‐old Friesian colt presented with severe, progressive distal limb lameness in two limbs. Radiographic and postmortem full‐body CT imaging revealed severe articular deforming osteolysis and osteoproliferation at the dorsodistal navicular bone margin and the adjacent distal phalanx proximo‐palmar/proximo‐plantar margin in two limbs, with the remainder of the distal interphalangeal joint being unaffected. Multiple other joints showed small osteolytic subchondral defects. Postmortem histopathological examination confirmed focal joint disease of both distal interphalangeal joints, without an identifiable cause. Based on clinical presentation, imaging findings, and histopathology, regional joint maldevelopment was suspected.

## 1 Signalment, History, and Clinical Findings

A 3‐month‐old Friesian colt (180 kg) was referred to Utrecht University Equine clinic for progressively worsening right front (RF) lameness. The onset of the lameness occurred 5 weeks prior to referral. No trauma or other relevant medical events were reported. The referring veterinarian treated the foal with Meloxicam 0.6 mg/kg q24 (Metacam 20 mg/mL) for 7 days, without clinical improvement of lameness.

Upon presentation at the Utrecht University Equine clinic, the foal was bright and alert with vital parameters within normal limits. Orthopedic examination revealed severe RF lameness graded 4/5 (AAEP scale). No heat, swelling, hoof deformity, or digital pulsation was observed, but a positive pain response to hoof tester pressure over the entire RF foot was noted. Bilateral RF perineural palmar digital nerve (PDN) anesthesia using 0.8 mL mepivacaine hydrochloride (Mepidor 20 mg/mL) significantly improved RF lameness. Blood analysis, including Serum Amyloid A concentration and hematology, was within normal limits. A presumptive diagnosis of a subsolar abscess was made, and treatment with a wet hoof bandage and Meloxicam 0.6 mg/kg q24 (Metacam 20 mg/mL) continued. The following day, left hind (LH) lameness graded 3/5 (AAEP scale) developed, while RF lameness remained unchanged. Re‐examination of the hooves revealed a similar positive pain response to pressure in the RF foot region. No abnormalities were noted in the LH limb.

Institutional animal care and use committee approval was not required because of the retrospective nature of this case study. Written informed consent was obtained from the owner of the animal, and anonymity is preserved.

## 2 Imaging, Diagnosis, and Outcome

Based on clinical findings and positive response to PDN anesthesia, the location of interest was determined to be the RF distal limb region. Because of the initial and longer‐existing RF lameness compared with LH, radiographic examination of the RF distal limb was performed first. Dorso 55° proximal‐palmarodistal oblique, lateromedial, dorsal 45° lateral‐palmaromedial oblique, and dorsal 45° medial‐palmarolateral oblique views of the proximal‐ and distal interphalangeal joint (PIPJ and DIPJ)—and metacarpophalangeal joint (MCPJ) were obtained (Canon‐Siemens Vertix Vet RF, Canon Medical Systems, Amstelveen, the Netherlands, and Siemens Healthineers, Den Haag, the Netherlands), and evaluated using image analysis software (Enterprise Imaging, Agfa Healthcare, Gent, Belgium).

**FIGURE 1:**
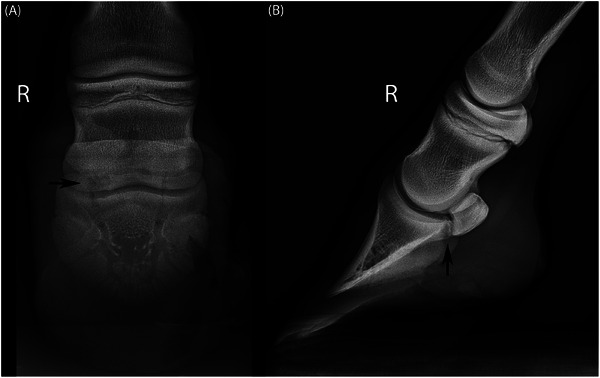
(A) Dorso 55° proximal‐palmarodistal oblique and (B) lateromedial radiographs of the distal right forelimb, lateral is to the left. There is an irregular radiolucent area and irregular bony extension at the lateral proximopalmar distal phalanx (arrow), which is in close contact with the distal navicular bone. Mild increased opacity in the adjacent subchondral bone of the distal phalanx and spongiosa of the navicular bone is noted.

Radiographs of the RF DIPJ revealed an ill‐defined, heterogeneous radiolucent zone on the lateral side of the proximopalmar surface of the distal phalanx (DP) (Figure 1A,B). The subchondral bone of the DP contained a focal indentation, with adjacent mild irregular and ill‐defined bony extension of the proximopalmar aspect of the DP, resulting in a relatively small distance between the navicular bone (distal sesamoid bone) and DP. The radiolucent lesion was superimposed on the navicular bone; therefore, involvement of the navicular bone could not be ruled out. Mild increased opacity of the surrounding bone of the DP and navicular bone spongiosa was present. Additionally, a small, irregular radiolucent subchondral border defect of the proximomedial articular surface of the proximal phalanx was visible.

Based on radiographic results, the primary finding was a focal osteolytic lesion of the palmar DP with possible sequestration. Due to its atypical location and clinical presentation, the differential diagnosis included a wide range of possible causes. Developmental diseases, such as osteochondrosis, traumatic subchondral osteonecrosis, or osteochondral fragmentation and osteomyelitis, possibly associated with (poly) arthritis, were considered, with the latter being less likely due to the lack of any clinically associated findings such as fever or inflammatory markers in the blood analysis.

Due to rapid clinical deterioration and poor prognosis, the owners of the foal declined further examination and opted for euthanasia. To further clarify radiographic findings, full‐body postmortem CT imaging was performed using a 64‐slice sliding gantry CT scanner (Somatom Definition AS, Siemens Healthineers), with the following settings 140 kVp, 360 and 400 mAs variable FOV to include all regions, 512 × 512 matrix, spiral pitch factor of 1, and reconstructed in bone algorithm (H60) with 2 mm slice thickness and soft tissue algorithm (H31) with 4 mm slice thickness. Sagittal multiplanar reconstruction of the lower limbs reconstructed in bone algorithm (H60) with 0.6 mm slice thickness and soft tissue algorithm (H31) with 1 mm slice thickness was also provided, and evaluated in the PACS.

**FIGURE 2:**
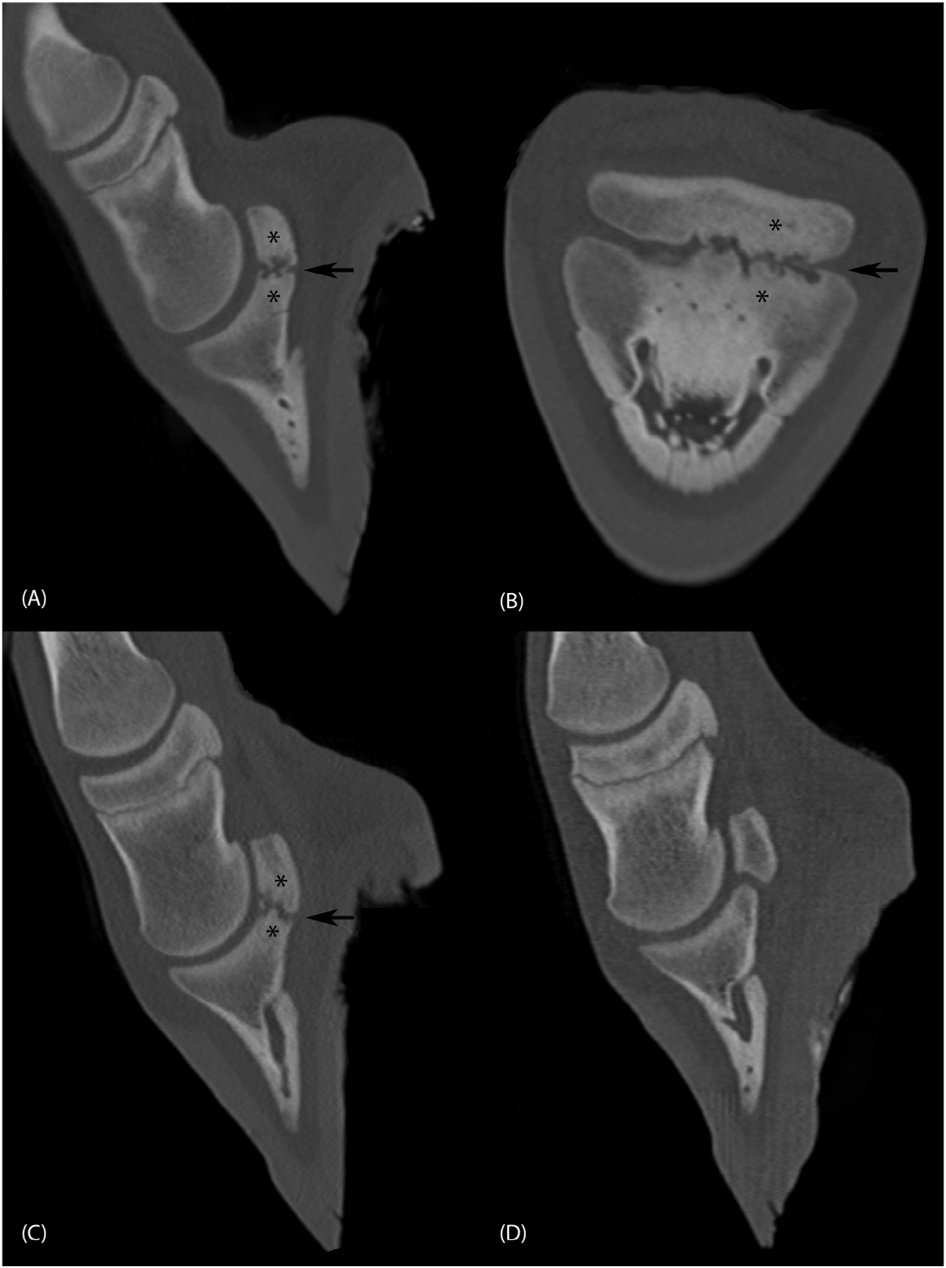
Multiplanar reconstruction (MPR) fan beam CT images with a bone algorithm of the distal limb. (A) Parasagittal and (B) dorsal plane of the left hind limb, lateral is to the right. Marked subchondral hypoattenuating bone lesions surrounded by a hyperattenuating area (asterisk) are visible at the level of the lateral articulation site of the navicular bone and the distal phalanx. Small, well‐defined, tubular hypoattenuating structures within the distal phalanx are visible as focal round regions in (B), likely representing vascular channels. There is an altered shape and elongation of the distal navicular bone and proximal distal phalanx, resulting in focal deformation and narrowing of the plantar joint space (arrow). (C) Parasagittal plane of the right front leg shows similar changes compared with the left hind (A), with a small intra‐articular mineral body. (D) Parasagittal plane of the left front limb at the same level as (C), showing normal morphology.

Comparable lesions were found in both the RF and LH limbs. The distal articular surface of the navicular bone and the corresponding palmar and, respectively, plantar, articular surface and subchondral bone of the DP showed a marked irregular bone structure and contour, extending from the mid‐sagittal plane to the lateral border (Figure 2A–C). Bony erosions or osseous cyst‐like osteochondral lesions were present as multiple ill‐defined, irregular hypoattenuating indentations. Concurrent bridging osteoproliferation extended from both the previously described regions of the DP and the navicular bone, resulting in a focal deformation of the navicular bone and extension of the proximopalmar and proximoplantar DP articular surface. Subsequent narrowing of the joint space with signs of a partial bony fusion of the articular surfaces was present. Small, well‐defined mineral bodies were present between the navicular bone and DP. Both the adjacent spongiosa of the navicular bone and the subchondral bone of the DP were markedly hyperattenuating. The remaining DIPJ was unaffected in both legs. In the right hind (RH) limb, one well‐defined focal hypoattenuating area surrounded by a hyperattenuating rim, resembling an osseous‐cyst‐like lesion, was found at the proximo‐plantar lateral parasagittal margin of the DP. The left front (LF) DIPJ and navicular bone appeared to be within normal limits (Figure 2D).

Additionally, small, well‐defined, subchondral defects with surrounding sclerosis were found in other joints of all legs except LF, in the following locations: RF proximal medial proximal phalanx, the LH distal sagittal proximal phalanx, the RH distal sagittal proximal phalanx and proximal sagittal middle phalanx, medial proximal third tarsal bone, proximal medial ridge of the talus, and axial medial femoral condyle. Given the presence of multiple additional smaller osteochondral lesions in other joints and considering the age and history of the foal, the imaging findings were most likely compatible with osteochondral defects.

**FIGURE 3:**
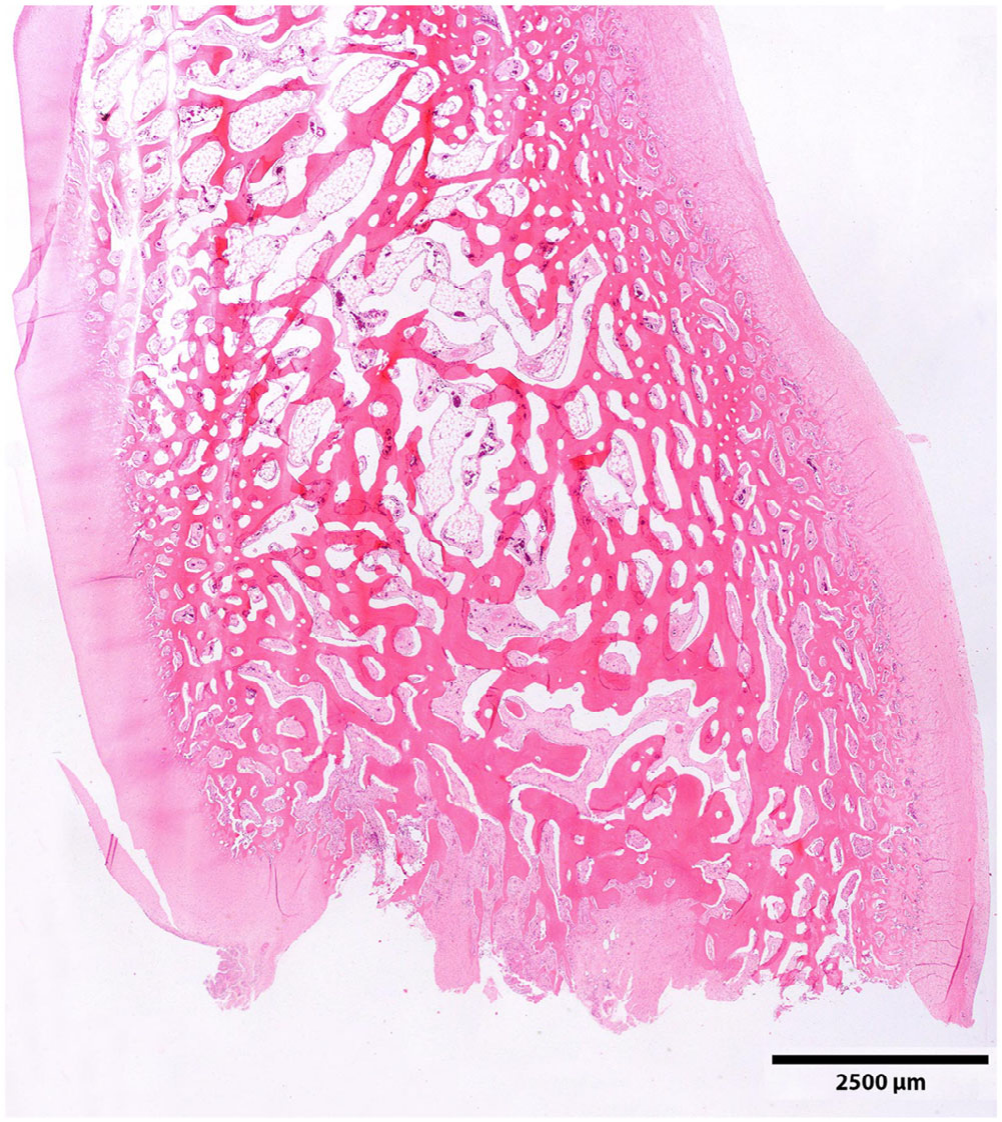
Sagittal tissue section of the right front navicular bone stained with hematoxylin and eosin; bar represents 2500 µm. Dorsal is to the left, distal at the bottom. A marked irregular distal border of the navicular bone is visible, with loss of hyaline cartilage, irregular compact bone trabeculae, and fibrous tissue extending into the spongiosa. There is widening of the bone trabeculae at the distal aspect of the navicular bone.

Postmortem macroscopic and histologic examination of the RF and LH navicular bone and DP was performed. On macroscopic evaluation, an irregular cartilage surface was seen at the RF and LH distal navicular bone and corresponding DP. Histologically, the RF and LH navicular bone contained a region characterized by an irregular articular surface consisting of fibrous tissue and irregular trabeculae of compact bone (Figure 3). The fibrous tissue extended into the underlying spongiosa, and the bony trabeculae showed marked thickening consistent with local myelofibrosis and osteosclerosis. In the areas covered with hyaline cartilage, no changes indicative of osteochondrosis were noted. Similar changes were appreciated in the corresponding DP, where the fibrous tissue contained large numbers of osteoclasts and macrophages with hemosiderin pigment. The joint capsule of RF and LH DIPJ showed moderate synovial proliferation and infiltration of lymphocytes. In conclusion, histopathological examination confirmed severe focal joint disease in both RF and LH DIPJ, without a histologically identifiable underlying cause.

## 3 Discussion

This case report documents a rare case of severe, two‐limb, deforming focal DIPJ disease in a foal. While distal limb radiographs revealed pathology involving the navicular bone and DP, full assessment of lesion extent changes was limited by superimposed distal limb bones. Computed tomography imaging proved valuable for defining the extent of bony lesions in both the navicular bone and DP, and additionally identifying subchondral, osteolytic lesions in other joints. On histopathology, severe focal joint disease was confirmed, although the exact pathogenesis remains unknown. The combination of early onset of pathology and advanced degenerative changes may have obscured early‐stage histological features such as ischemic chondronecrosis, typical of osteochondrosis [1].

Notably, the location of pathology was confined to the lateral half of the distal navicular bone and proximal DP. Comparable imaging features have been reported in nine calves aged 2 to 6 months, where radiographic and CT imaging revealed subchondral bone lysis and bridging osteoproliferation of the palmar and plantar side of the DIPJ in multiple limbs [2]. Postmortem histology could not determine an underlying cause; however, developmental disease, such as joint hypoplasia or osteochondrosis, was suspected. Similar radiographic findings, including joint space narrowing and subchondral bone lysis of the DIPJ, were reported in two calves, which eventually led to complete ankylosis in multiple limbs without an assigned cause [3]. In our case, early detection and rapid progression may explain the focal changes without widespread joint disease.

Congenital maldevelopment or dysplasia of the DIPJ is considered the most likely cause of severe joint disease in this foal, supported by similar lesions in multiple limbs and young age. Several case reports of navicular bone and/or DP aplasia or hypoplasia in foals are available [4, 5, 6, 7, 8]. In all described cases, foals show marked lameness and abnormal limb conformation. In 17 of 21 joints of calves with similar imaging findings as this case, the navicular bone was displaced axially and smaller in size [2], indicating navicular bone hypoplasia. One foal showed suspected congenital fusion of the navicular bone and DP, with an overall decrease in size of the bones [9]. Additionally, dysgenesis of regional soft tissue structures such as the navicular bursa, deep digital flexor tendon, vasculature, and joint structures is described, with one case describing the absence of articular cartilage and DIPJ extension between the navicular bone and DP [4, 5]. In our case, bone size reduction or conformation abnormalities were not present; however, localized dysplasia of the DIPJ could be considered as an underlying cause of abnormal development of the joint surface.

Another more common developmental joint disease in young horses is osteochondrosis, which involves a failure in endochondral ossification due to vascular disturbances in growth cartilage, potentially leading to degenerative joint disease [10]. Although no histopathological exam was performed on the remaining joints, multifocal osteochondral lesions might indicate multifocal osteochondral development disorder. While the palmar or plantar DIPJ is an atypical site for osteochondrosis, a recent case series including four horses documented osteochondral fragmentation of the palmarolateral/plantarolateral aspect of DP, with osteochondrosis histologically confirmed in one case [11]. In our case, the pathology occurred in a similar location, but subchondral bone lysis and bony proliferation were more severe.

While developmental diseases, such as osteochondrosis or congenital joint dysplasia, were considered most likely underlying pathology, other causes might have contributed to the severity of focal joint disease. One possible cause of secondary degenerative joint disease is subchondral bone osteonecrosis, which can occur due to failure of vascularization of the subchondral bone. Subchondral bone osteonecrosis can lead to articular cartilage collapse and secondary degenerative joint disease, as seen in the femoral head of young dogs [12]. In horses, abnormal vascular supply of the navicular bone during fetal development has been linked to the development of tri‐ and bipartite navicular bones [13]. Radiographic and histological examination of tri‐ and bipartite navicular bones reveals degenerative lesions with both subchondral cysts and sclerosis at the region of separation, comparable to our imaging findings. Interestingly, a study on navicular bone vascular patterns revealed specific regions prone to partition due to blood supply from a singular direction [14]. The parasagittal distribution of lesions in this case report may correlate with such a specific vascular pattern in localized joint disease.

Although trauma‐induced joint disease was considered unlikely, abnormal mechanical loading from joint maldevelopment or instability could have caused secondary degenerative changes. Postnatal ossification of the navicular bone and DP occurs in foals, leading to changes in the size and shape of the articular surfaces [15, 16, 17]. Therefore, early disturbance of ongoing ossification might explain the rapid progression of lesions in this area. As mentioned earlier, malalignment secondary to navicular bone displacement might have contributed to DIPJ ankylosis in calves [2]. Besides pathological bony alignment, abnormal mechanical forces in the navicular region can also be linked to adjacent tendons and ligaments [18]. In Friesian horses, an altered collagen turnover and biochemical composition of tendon tissue have been reported, which could potentially affect biomechanical properties [19, 20]. In this case, no histologic examination of the ligaments in the navicular apparatus was performed, but no macroscopic abnormalities were observed.

In conclusion, two‐limb congenital partial fusion of the navicular bone and DP with severe focal bone modelling and multifocal developmental osteochondral disease was identified in a progressively lame 3‐month‐old Friesian foal. Most likely, localized joint dysplasia, potentially combined with osteochondrosis of the palmaro‐/plantarolateral side of DP, aberrant vascularization, and mechanical stress contributed to the severe joint disease. Histopathology confirmed secondary severe joint disease with extensive osteochondral modelling. Full‐body CT imaging was very valuable in identifying the full extent of lesions across the affected joints and may aid in the clinical work‐up of multilimb lameness in juvenile equine patients.

## Author Contributions

### Category 1

- Conception and design: Giessen
- Acquisition of data: Veraa, Stas, Grinwis
- Analysis and interpretation of data: Giessen, Veraa, Stas, Grinwis

### Category 2

- Drafting the article: Giessen
- Reviewing article for intellectual content: Veraa, Stas, Grinwis

### Category 3

- Final approval of the completed article: Giessen, Veraa, Stas, Grinwis

### Category 4

  - Agreement to be accountable for all aspects of the work in ensuring that questions related to the accuracy or integrity of any part of the work are appropriately investigated and resolved: Giessen, Veraa, Stas, Grinwis

## Conflicts of Interest

The authors declare no conflicts of interest.

## Previous Presentation or Publication Disclosure

The authors have nothing to report.

## Reporting Checklist Disclosure

No reporting checklist was used.

## Data Availability

The data that support the findings of this study are available from the corresponding author upon reasonable request.
